# Mesenchymal stem cells combined with liraglutide relieve acute lung injury through apoptotic signaling restrained by PKA/β-catenin

**DOI:** 10.1186/s13287-020-01689-5

**Published:** 2020-05-19

**Authors:** Xiaotong Yang, Xiaoying Ma, Ocholi Don, Yuanlin Song, Xiaoyan Chen, Jianwen Liu, Jieming Qu, Yun Feng

**Affiliations:** 1 Department of Respiratory and Critical Care Medicine, Ruijin Hospital, School of Medicine, Shanghai Jiao Tong University, Shanghai, 20025 China; 2 State Key Laboratory of Bioreactor Engineering & Shanghai Key Laboratory of New Drug Design, School of Pharmacy, East China University of Science and Technology, Shanghai, 200237 China; 3 Department of Pulmonary Medicine, Zhongshan Hospital, Fudan University, Shanghai, 20003 China; 4 Department of Pathology, Ruijin Hospital, School of Medicine, Shanghai Jiao Tong University, Shanghai, 20025 China; 5 Institute of Respiratory Diseases, School of Medicine, Shanghai Jiao Tong University, Shanghai, 20025 China

**Keywords:** ARDS, ALI, Liraglutide, Mesenchymal stem cells, Cell apoptosis

## Abstract

**Background:**

ARDS and ALI are life-threatening diseases with extremely high mortality in patients. Different sources of MSCs could mitigate the symptoms of ALI from diverse mechanisms. Liraglutide is an activator of glucagon-like peptide-1 receptor (GLP-1R) that activates anti-apoptotic pathways and exerts anti-inflammatory effects. We mainly compared the effects of human chorionic villus-derived mesenchymal stem cells (hCMSCs), human bone marrow-derived mesenchymal stem cells (hBMSCs), and human adipose-derived mesenchymal stem cells (hAMSCs) on the treatment of ALI and explored the apoptosis mechanism of combination MSCs of liraglutide.

**Methods:**

The proliferation of MSCs was detected by MTT assay. Western blot and RT-qPCR were used to detect the expression of GLP-1R, SPC, Ang-1, and KGF in MSCs stimulated by LPS and liraglutide. By using flow cytometry and TUNEL assay to compare the apoptosis of three MSCs under the action of LPS and liraglutide, we selected hCMSCs as the target cells to study the expression of apoptotic protein through the PKA/β-catenin pathway. In ALI animal models, we observed the effects of liraglutide alone, MSCs alone, and MSCs combined with liraglutide by H&E staining, cell counting, immunohistochemistry, and ELISA assay.

**Results:**

We demonstrated that LPS attenuates the proliferation of the three MSCs and the expression of GLP-1R. Liraglutide could reverse the effects of LPS; increase the expression of SPC, Ang-1, and KGF; and can reduce the apoptosis of three MSCs through the PKA/β-catenin pathway. In the LPS-induced ALI model, MSCs combined with liraglutide showed a significant therapeutic effect, and hCMSCs combined with liraglutide have advantages in the treatment of ALI.

**Conclusions:**

The therapeutic effect of combination MSCs of liraglutide on ALI was higher than that of MSCs alone or liraglutide alone, and liraglutide could alleviate the symptoms of ALI by reducing MSCs apoptosis.

## Background

In critically ill patients, acute lung injury (ALI) and acute respiratory distress syndrome (ARDS) are the leading causes of acute respiratory failure and have a high mortality ratio [1]. The characteristics of ARDS and ALI are mainly the complex relationship between the immune system and alveolar capillaries, leading to acute pro-inflammatory reactions [2], accompanied by increased pulmonary protein permeability, enhanced pulmonary edema, slowed alveolar clearance, and ultimately impaired gas exchange and hypoxemia [3–5]. The current main therapeutic strategies are supportive treatments [6], including lung-protective ventilation [7], prone positioning [8], early neuromuscular blockade [9], and conservative fluid management [10]. However, the therapeutic effect was not significantly improved [7], and effective treatment strategies are urgently required. Lipopolysaccharide (LPS) has been identified as a cause of ALI [11], which could directly damage endothelial cells and indirectly activates neutrophils and macrophages to exert toxic effects on pulmonary [12].

Recently, using of mesenchymal stem cells (MSCs) in the treatment of ALI has proven to be effective [13]. They secrete paracrine factors that regulate lung endothelial and epithelial permeability, including growth factors, antimicrobial peptides, and anti-inflammatory cytokines, which could alleviate damaged alveolar fluid clearance, improve lung permeability, and mitigate inflammatory disorders [14–18]. In addition, MSCs are widely available and can be collected from the bone marrow [19], adipose tissues [20], placenta [21], umbilical cord [22], and other tissues [23, 24]. However, inflammatory stimulation in the ALI lung microenvironment could lead to a decrease in MSC survival and weaken paracrine capacity [24]. Several studies have been reported to identify strategies to ameliorate and improve the efficiency of MSC therapy [13, 14, 25].

Glucagon-like peptide-1(GLP-1) is an endogenous 30-amino acid peptide synthesized by enteroendocrine L cells and secreted throughout the small intestine and ascending colon which influence the absorption of nutrients [26]. Liraglutide is a long-acting glucagon-like peptide analog that targets the GLP-1 receptor (GLP-1R) [27]. Studies have shown that liraglutide has a good anti-inflammatory function and could regulate the inflammatory response to limit the progression of atherosclerosis in vivo [28]. In addition, it was reported that liraglutide could treat myocardial infarction by increasing myocardial blood flow, inhibiting cardiomyocyte apoptosis, and enhancing cardiac function [29]. It has also been found in our previous research that liraglutide can be combined with MSCs to alleviate the symptoms of ALI [30].

Based on above investigation, we aimed to contrast the comparison of human chorionic villus-derived mesenchymal stem cells (hCMSCs), human bone marrow-derived mesenchymal stem cells (hBMSCs), and human adipose-derived mesenchymal stem cells (hAMSCs) under the action of LPS and liraglutide. The protective effects of three MSCs combined with liraglutide on ALI animal models were examined simultaneously. Furthermore, we assessed the anti-apoptotic effect of liraglutide on MSCs by activating the PKA/β-catenin pathway to suppress the Bax, Bcl-2, cleaved caspase-9, and cleaved caspase-3 apoptotic signaling pathways.

## Methods

### Cell culture and transfection

hCMSCs and hBMSCs were characterized by staining with antibodies against CD44, CD73, CD90, CD105, CD34, and CD45 and then detected by flow cytometry as described in our previous study [31]. hAMSCs were kindly provided by Dr. Chuandong Wang (Department of Orthopedic Surgery, Xin Hua Hospital Affiliated to Shanghai JiaoTong University School of Medicine, Shanghai, China) and were verified by staining with antibodies against CD34, CD45, CD73, CD90, and CD105 and then detected by flow cytometry as described in Wang’s article [32]. MSCs were cultured in mesenchymal stem cell medium (ScienCell, Cat. No. 7501, USA) in a humidified atmosphere at 37 °C and 5% CO_2_. MSCs were harvested at approximately 80–90%, and the culture medium was changed every 2–3 days.

Cells were seeded onto desired size plates to reach 70–90% and then transfection was performed using Lipofectamine™ 2000 (Thermo Fisher Scientific, 11668027, USA) according to the manufacturer’s protocol. The si-GLP-1R were synthesized by Hanbio Biotechnology Co., Ltd. (Shanghai, China). The sequences are as follows:

Hs-GLP-1R-si-1

5′-GGAAGACUGUCAACACUAAdTdT-3′; 5′-UAGAAAUCUAUCUUUGUCCdTdT-3′;

Hs-GLP-1R-si-2

5′-GAAUAGUCUGUGUGCACAAdTdT-3′;5′-UUGUGCACACAGACUAUUCdTdT-3′;

Hs-GLP-1R-si-3

5′-GGAAGGAUGUGCUUUCCUAdTdT-3′; 5′-UAGGAAAGCACAUCCUUCCdTdT-3′;

Negative control (NC)

5′-UUCUCCGAACGUGUCACGdTdT-3′; 5′-ACGUGACACGUUCGGAGAAdTdT-3′.

The Si-GLP-1R silencing efficiency was determined 48 h post transfection by protein and mRNA analysis for future experiments.

### MTT assay

MTT (3-(4,5-dimethylthiazol-2-yl)-2,5-diphenyltetrazolium bromide) assay was used to detect cell viability of three different MSCs. The three MSCs were at 1 × 10^4^ cells/well in 96-well plates and then divided into three groups: (i) no treatment (control group), (ii) treated with 30 μg/mL LPS (L2880, Sigma, USA) (LPS group), and (iii) treated with 30 μg/mL LPS and 10 nM liraglutide (MK, Cat. No. 204656-20-2, China) (LPS + liraglutide group). The concentrations of LPS and liraglutide were selected based on previous studies of MSCs viability [30]. After 6, 24, 48, and 72 h of incubation, 10 μL of 5 mg/mL MTT (M2128, Sigma, USA) solution was added to each well and continued to culture at 37 °C for 4 h. Cells were lysed using 100 μL of dimethylsulfoxide (DMSO, 67-68-5, Sigma, USA). The optical density (OD) was measured at both 492 nm and 630 nm wavelength. Date represents the mean of four wells at each point.

### Western blot assay

To verify the expression of KGF, SPC, GLP-1, and GLP-1R, MSCs were exposed to 30 μg/mL LPS or 10 nM liraglutide. To verify the PKA/β-catenin pathway, MSCs were exposed to 10 nM liraglutide or 100 nM SiRNA or 20 μM PKA inhibitor H89 (MCE, Cat. No. 130964-39-5, USA) on the premise of exposure to 30 μg/mL LPS. Proteins were extracted from differently treated MSCs. The protein concentration was determined using the BCA assay (CST, #7780, USA). Proteins were separated on SDS-PAGE gel ahead of being transferred on PVDF membranes (Life Technologies, USA). The PVDF membrane was blocked in 5% BSA solution for 3 h, after which the different primary antibodies were applied overnight including Angiopoietin 1 (Ang-1, Abcam, ab102015,UK), GLP-1R (Novus, NBP1-97308, USA), SFTPC (SPC, Abclonal, A1835, USA), KGF (Abcam, ab131162 UK), p-β-catenin (Santa Cruz, sc-57535, USA), β-catenin (Wanleibio, WL0962a, China), cleaved caspase-3 (Abcam, ab49822,UK), cleaved caspase-9 (Abways, CY5682, Chian), Bax (Wanleibio, WL03446, China), and Bcl-2 (Abways, CY6717, China). GAPDH (Santa Cruz, sc-166574, USA) was used as a loading control of total protein. The membranes were then incubated with HRP secondary antibody (Jackson ImmunoResearch Laboratories, 111-035-003, 115-035-003, China). The blots were detected by ECL technique.

### RT-qPCR assay

Total RNA was extracted from three different MSCs using TRIzol reagent (Invitrogen, 15596-026, USA). TransScript First-Strand cDNA Synthesis SuperMix (Transgene Biotech, AH341-01, China) was used for cDNA synthesis following the manufacturer’s instruction. The RT-qPCR was carried out on an CFX96 (Bio-Rad, USA) and TransStart Top Green qPCR SuperMix (Transgene Biotech, AQ131-01, China) with a gene-specific primer (Table 1). The relative expression of gene was expressed as a function of threshold cycle (Ct) and analyzed by 2^-ΔΔCt^ method.

**Table 1:** The sequence of primers

Gene	Primers
GLP-1R	F:5′- ATCACAGTGGCGAGAGGAGAG-3′ R: 5′-CCAAGTGATGCAAGCAGAGG-3′
SPC	F: 5′-GGGTCATCCAGGCAACTCGG-3′ R: 5′-CTTTCCCGTTGGGGCTTCC-3′
KGF	F: 5′-GACAGCAGACACGGAACTCT-3′ R: 5′-AGTCGTCGCTCTTTCCAAACT-3′
Ang-1	F: 5′-TGGCTTGGATGTGCAACCTT-3′ R: 5′-CCCCCTCAAAGAAAGCGTTTG-3′
GAPDH	F:5′-TGCCAAATATGATGACATCAAGAA-3′ R:5′- GGAGTGGGTGTCGCTGTTG-3′

### Immunofluorescence staining

The 1 × 10^5^ MSCs were plated in a 24-well plate, after treatment with LPS and liraglutide, hCMSCs, hBMSCs, and hAMSCs were fixed in 4% paraformaldehyde (solarbio, P1110, China) at 4 °C for 20 min, washed with 0.1% BSA in PBS, and then permeabilized with 0.3% Triton× 100 (Sigma, 9002-93-1, USA) at room temperature. The cells were incubated with anti-GLP-1R antibody at 5 μg/mL overnight at 4 °C and detected with anti-rabbit Cy3 (Jackson ImmunoResearch, 111-165-003, China) (red) 1:100 dilution. Nuclei were stained with DAPI (Sigma, 28718-90-3, USA) (blue). MSCs were examined under a fluorescence microscope (Nikon, Japan). MSCs were imaged using a × 10 objective. Randomly, they selected 8 fields of view for quantitative fluorescence analysis.

### Immunohistochemistry analysis

The pulmonary tissue sections were incubated at 60 °C for 4 h and immersed in xylene twice for 20 min each, rehydrated by means of a graded series of ethanol, and then incubated with fresh 0.3% hydrogen peroxide in 100% methanol for 30 min at room temperature. The sections were then washed three times using PBS for 15 min. Antigen retrieval was performed with the ImmunoSaver antigen retriever system (Boster, AR0023, USA) at 98–100 °C for 30 min, and sections were cooled to room temperature. Sections were then washed by PBS. Nonspecific binging sites were blocked by incubation with BSA solution (Boster, SA1022, USA) for 30 min. The sections were then incubated with SFTPC antibody (Abclonal, A1835, USA) or Nanog antibody (Thermo Fisher Science, PA1-097, China) in a 1:100 dilution overnight at 4 °C and incubated with secondary antibodies at room temperature for 30 min. The sections were performed according to the steps of the SABC immunohistochemical staining kit (Boster, SA1022, USA). The sections were imaged using a × 40 objective.

### TUNEL analysis

The TUNEL experiment (Beyotime, C1088, China) follows the instructions. The 1 × 10^5^ MSCs were plated in a 24-well plate, divided into control groups, 30 μg/ml LPS groups and 30 μg/mL LPS combined with 10 nM liraglutide groups, and cultured for 72 h. The MSCs were washed once with PBS, then fixed in 4% paraformaldehyde (solarbio, P1110, China) for 30 min at 4 °C, and later incubated with 0.3% Tritonx-100 (Sigma, 9002-93-1, USA) for 5 min at room temperature. After washing twice using PBS, the TUNEL solution (Beyotime, C1088, China) was added to each well and incubated at 37 °C for 60 min in the dark. Afterwards, it was washed 3 times with PBS. DAPI (Sigma, 28718-90-3, USA) was incubated the nuclei in the dark at room temperature for 10 min. MSCs were imaged using a × 20 objective.

### Flow cytometric analysis

MSC apoptosis were performed by an Annexin V-FITC/propidiumiodide (PI) kit (4A Biotech, FXP018, China). MSCs were collected using different treatments and prepared 50 μL of a 2 × 10^5^ cell suspension in PBS. The MSCs were then incubated with Annexin V-FITC and PI for 5 min at room temperature and analyzed using a FACS Vantage cytometer (Becton Dickinson, USA). The proportion of apoptotic cells was analyzed by FlowJo software.

### In vivo modeling

**Fig. 1:**
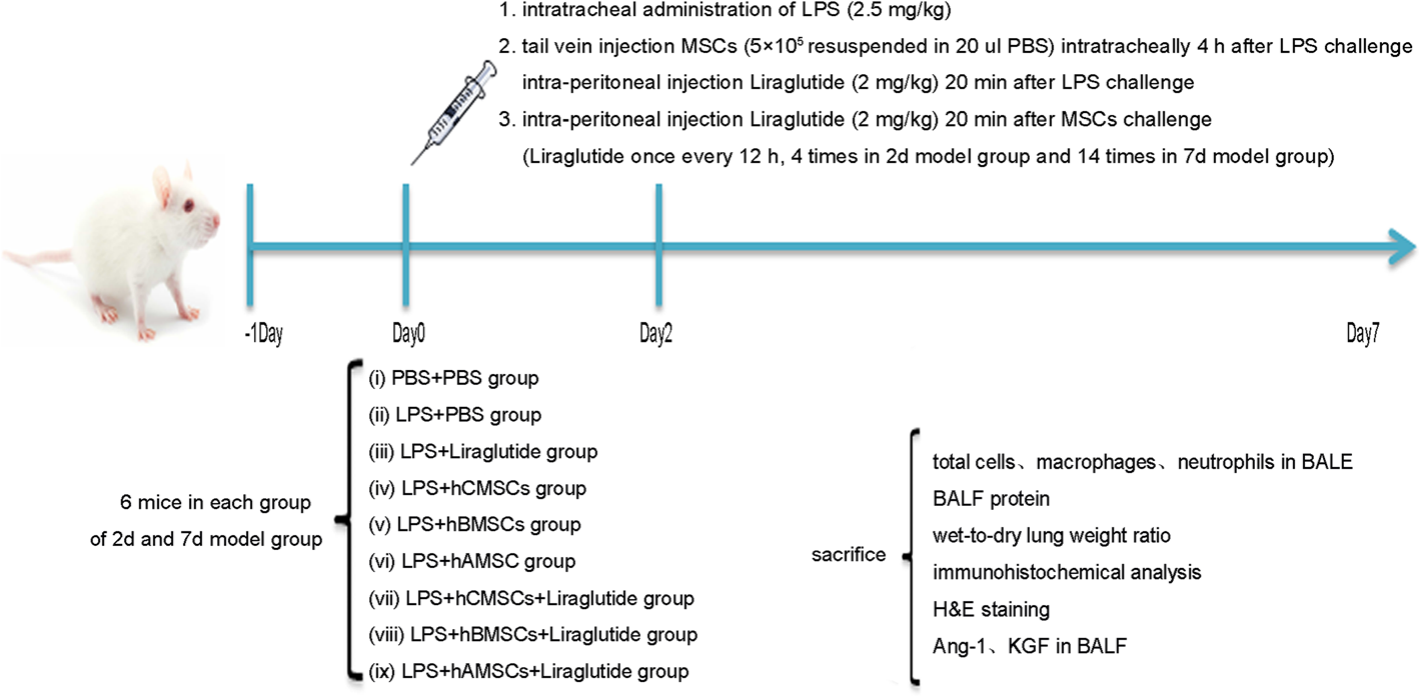
Animal experiment flow chart. Human chorionic villus-derived mesenchymal stem cells (hCMSCs), human bone marrow-derived mesenchymal stem cells (hBMSCs), human adipose-derived mesenchymal stem cells (hAMSCs), and bronchoalveolar lavage fluid (BALF)

Four-week-old male SPF BALB/C mice were purchased from Cavensbiogle (Suzhou, China). The mice were kept in a conducive environment and, within a week, had adapted to it and able to freely access food and water. Mice were originally divided into nine groups (twelve mice per group): (i) PBS + PBS group, (ii) LPS + PBS group, (iii) LPS + liraglutide group, (iv) LPS + hCMSCs group, (v) LPS + hBMSCs group, (vi) LPS + hAMSCs group, (vii) LPS + hCMSCs+Liraglutide group, (viii) LPS + hBMSCs + liraglutide group, and (ix) LPS + hAMSCs + liraglutide group. After LPS stimulation, six mice were randomly selected from each group and classified into the 2d model group and 7d model group. In the LPS + PBS group, mice were slowly instilled via the trachea with 2.5 mg/kg LPS (dissolved in 50 μL of 0.9% normal saline). The PBS + PBS group were injected with the same volume of 0.9% normal saline. The LPS + liraglutide group was injected with the same volume of LPS, and 20 min later, it was intraperitoneally injected 2 mg/kg liraglutide every 12 h. The LPS + hCMSCs group, LPS + hBMSCs group, and LPS + hAMSCs group were injected with the same volume of LPS which then 4 h later, 200 μL PBS containing 5 × 10^5^ different MSCs were injected into the tail vein. The LPS + hCMSCs + liraglutide group, LPS + hBMSCs + liraglutide group, and LPS + hAMSCs + liraglutide group were treated the same way as the LPS + hCMSCs group, LPS + hBMSCs group, and LPS + hAMSCs group. After 20 min of treatment, 2 mg/kg of liraglutide was intraperitoneally injected every 12 h (4 times in 2d model group and 14 times in 7d model group) [33]. All animals were humanely killed by lethal overdose of sodium pentobarbital in 2d and 7d model group after LPS stimulation. The animal experiment flow chart is shown in Fig. 1.

### H&E staining

After 2d or 7d of LPS stimulation, mice were sacrificed and their pulmonary tissues were fixed in 4% paraformaldehyde overnight, embedded in paraffin, sectioned into 3–5 μm thickness, and stained with hematoxylin and eosin (H&E, Cat. No.C0105, Beyotime). The pathological changes of pulmonary tissues were observed under a microscope using × 20 objective. According to the pulmonary tissue pathological damage score [34], the researchers were blinded to the group and randomly selected 10 fields of view in the × 400 field of view, with each variable being scored with 0 to 4 points. The scoring criteria were as follows: no injury scored 0, injury in 25% of the field scored 1, injury in 50% of the field scored 2, injury in 75% of the field scored 3, and injury throughout the field 4.

### Total protein concentration and inflammatory cell counts in the BALF

After 2d or 7d of LPS stimulation, we collected the bronchoalveolar lavage fluid (BALF), and the supernatant was centrifuged to determine the total protein concentration by the BCA protein kit (CST, #7780, USA). The total number of nucleated cells in the BALF was counted using a hemocytometer. The pellet was then resuspended in 200 μL PBS. Ten microliters of cell suspension was added to the coverslip and stained with Wright-Giemsa staining solution (Solarbio, G1020, China); 200 cells were counted under 8 randomly selected fields using a microscope of × 100 magnification.

### Wet-to-dry ratio (W/D)

After 2d or 7d of LPS stimulation, mice were sacrificed and their lungs were collected. The severity of pulmonary edema was assessed by the wet-to-dry ratio (W/D). The left lower lung was weighted and then dehydrated at 60 °C for 72 h in an oven.

### ELISA assay

In vitro, three kinds of MSCs were seeded with 30 μg/mL LPS alone or with 10 nM liraglutide after 72 h. The culture media were collected and detected for the secretion of IL-10 (Neobioscience, Cat. No. EMC005, China) and TGS-6 (RayBio, Cat. No. P98066, China). The BALF collected and detected the levels of Ang-1 (Dakewe, Cat. No. 100-170-APH, China) and KGF (Dakewe, Cat. No. KOA0490, China) following the instruction of the ELISA kits.

### Statistical analysis

All experiments were repeated at least three times. Quantitative results were expressed as the means ± standard deviation (SD). The comparison between SiRNA-control and SiRNA-GLP-1R was examined using the Student’s *t* test with SPSS 22.0 software (IBM Corp., Armonk, NY, USA). Comparisons among multiple groups were performed using one-way analysis of variance (ANOVA) with Dunnett’s post hoc test and Mann-Whitney test using GraphPad Prism 5.02 software. *P* < 0.05 was considered to indicate a statistically significant difference.

## Results

### Expression of GLP-1R in three MSCs at different time points stimulated by LPS

**Fig. 2:**
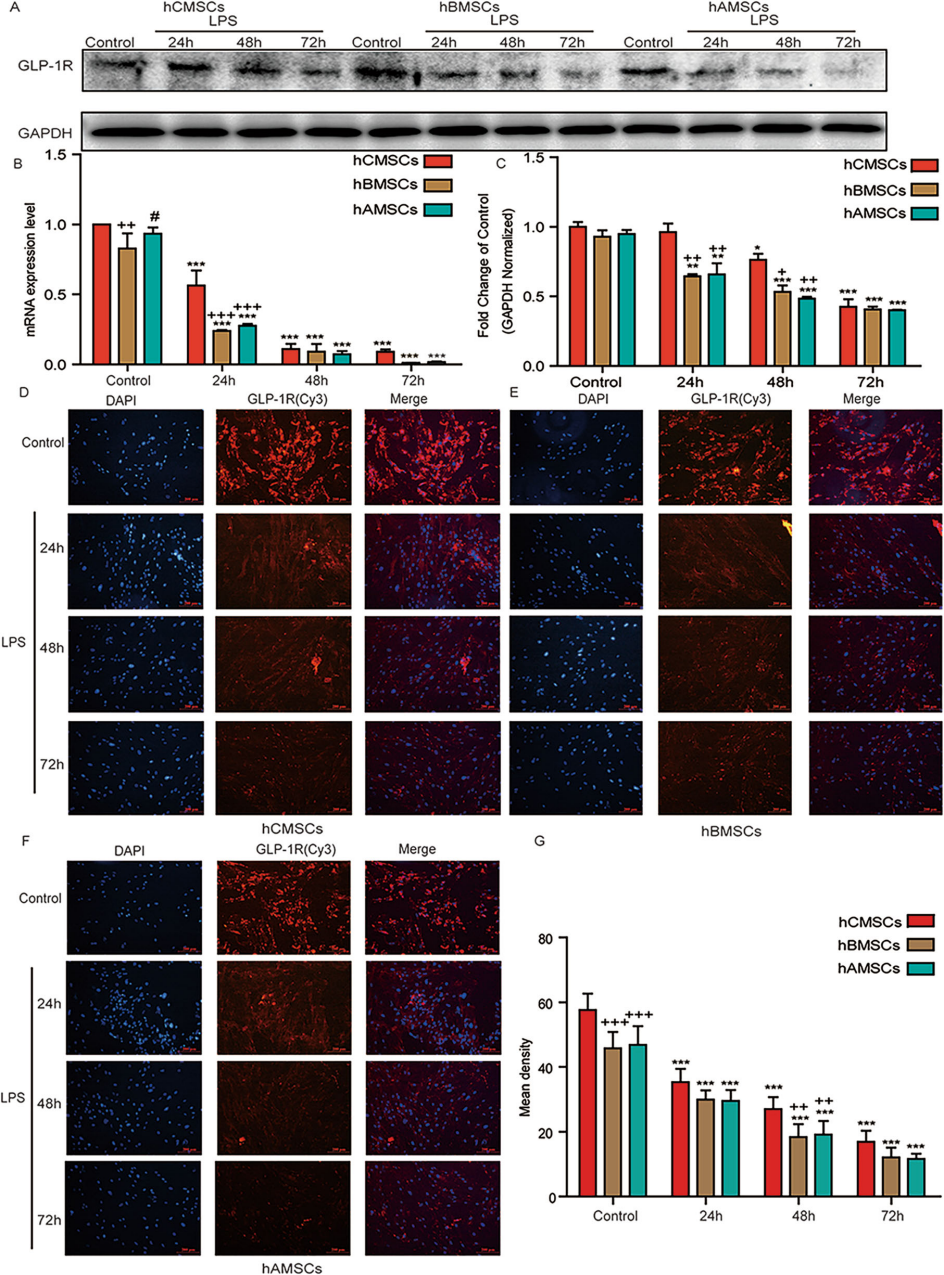
Expression of GLP-1R in three MSCs at different time points under the stimulation of LPS. **a**–**c** The expression of GLP-1R in hCMSCs, hBMSCs, and hAMSCs in the control group and in 30 μg/mL LPS stimulation for 24 h, 48 h, and 72 h at protein or mRNA level. Fluorescence intensity of GLP-1R in hCMSCs (**d**), hBMSCs (**e**), hAMSCs (**f**), and mean intensity (**g**) in the control group and 30 μg/mL LPS stimulation for 24 h, 48 h, and 72 h. Blue stands for DAPI and red stands for GLP-1R protein. The representative pictures were showed at × 10 original magnification. Scale bars, 200 μm, and error bar represent mean ± SD from three independent experiments. Compared with the control group corresponding to MSCs, *P < 0.05, **P < 0.01, and ***P < 0.001; compared with the hCMSCs group corresponding to time points, ^+^*P* < 0.05, ^++^*P* < 0.01, and ^+++^*P* < 0.001; compared with the hBMSCs group corresponding to time points, ^#^*P* < 0.05

Figure 2 showed the expression of GLP-1R in three MSCs under LPS stimulation. The protein expression of GLP-1R in three MSCs was analyzed by western blot and it decreased gradually with the increase of LPS stimulation time (Fig. 2a). Correspondingly, the ratio of GLP-1R/GAPDH was significantly reduced (Fig. 2b). The level of GLP-1R in hCMSCs was higher than that of hBMSCs and hAMSCs. GLP-1R at mRNA level was detected by RT-qPCR (Fig. 2c). The results showed that the level of GLP-1R was decreased under the stimulation of LPS, and it was higher in hCMSCs. The fluorescence area and mean intensity were showed the same results (Fig. 2d–g).

### Expression of Ang-1, SPC, KGF, and cytokines and proliferation of three MSCs under the stimulation of LPS and liraglutide

**Fig. 3:**
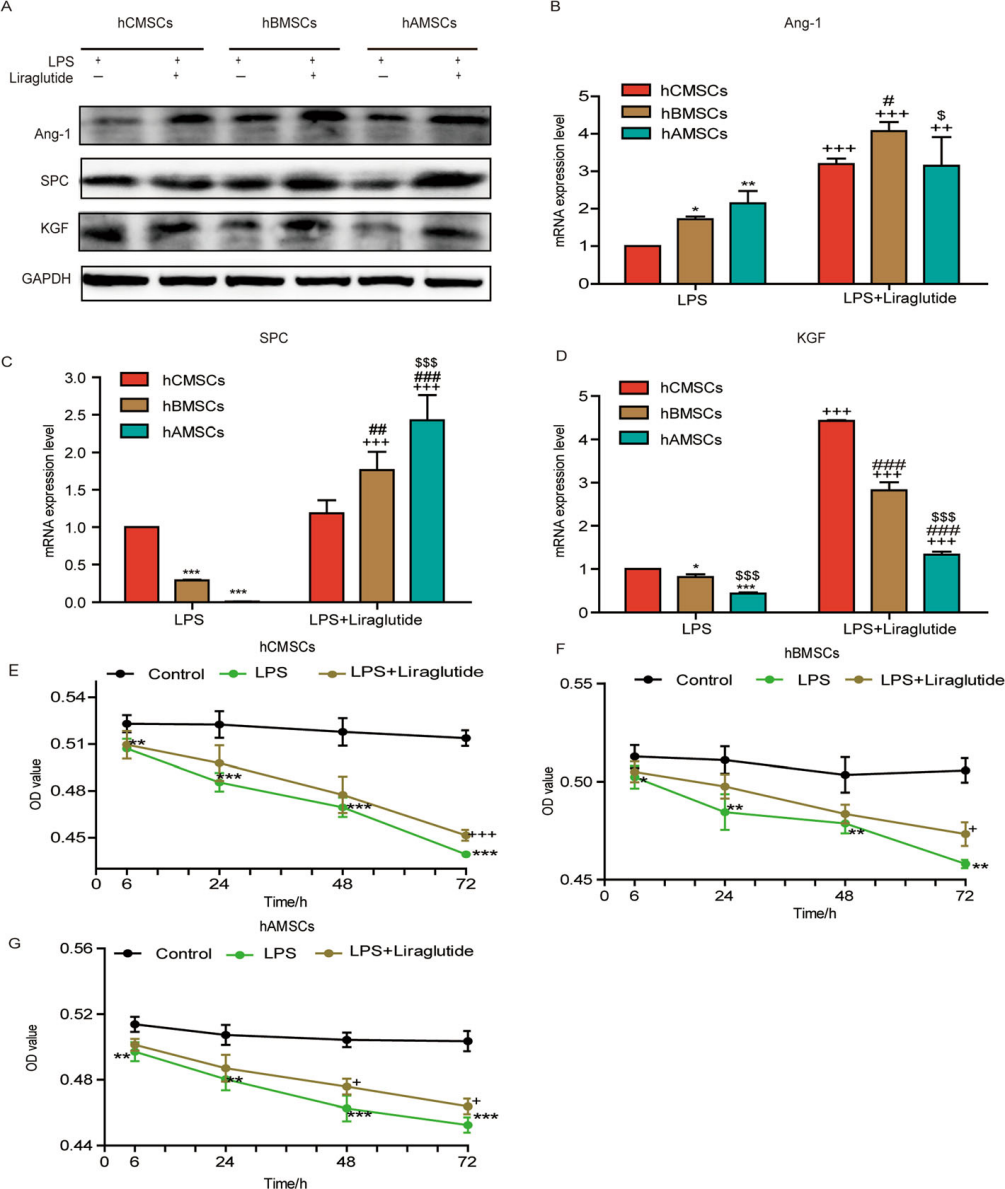
The expression of Ang-1, SPC, and KGF under the stimulation of LPS alone and combination of liraglutide for 72 h in three MSCs. **a** Ang-1, SPC, KGF, **b** Ang-1, **c** SPC, and **d** KGF are expressed at protein or mRNA level under the stimulation of 30 μg/mL LPS alone and combination of 10 nM liraglutide at 72 h. Error bars represent mean ± SD from three independent experiments. Compared with the hCMSCs group stimulated by LPS, *P < 0.05, **P < 0.01, and ***P < 0.001; compared with the LPS group corresponding to MSCs, ^++^*P* < 0.01 and ^+++^*P* < 0.001; compared with the hCMSCs group stimulated by LPS and liraglutide, ^#^*P* < 0.05, ^##^*P* < 0.01, and ^###^*P* < 0.001; compared with the hBMSCs group stimulated by LPS and with or without liraglutide, ^$^*P* < 0.05 and ^$$$^*P* < 0.001. The survival rate of hCMSCs (**e**), hBMSCs (**f**), and hAMSCs (**g**) in the control group, LPS group, and LPS + liraglutide group. Error bars represent mean ± SD from three independent experiments. Compared with the control group corresponding to time points, *P < 0.05, **P < 0.01, and ***P < 0.001; compared with the LPS group corresponding to time points, ^+^*P* < 0.05, ^+++^*P* < 0.001

Under the stimulation of LPS, the levels of cytokines IL-10 and TNF-α-stimulated gene/protein 6 (TSG-6) were reduced, and the degree of inflammation is enhanced. Under the action of liraglutide, the levels of IL-10 and TSG-6 were increased, which reduces the level of inflammation (Additional file 1). To verify whether liraglutide could raise the expression of Ang-1, SPC, and KGF at protein or mRNA level, western blot and RT-qPCR were used to detect the expression at 72 h (Fig. 3a–d). The expression of KGF was higher in hCMSCs, the expression of Ang-1 was higher in hBMSCs, and the expression of SPC was more in hAMSCs. In order to compare the proliferation of three MSCs under the action of LPS and liraglutide, we observed the growth state of three MSCs by MTT assay. Under the stimulation of LPS, the proliferation of three MSCs decreased at 6, 24, 48, and 72 h. Conversely, liraglutide could significantly enhance MSC growth under LPS stimulation (Fig. 3e–g).

### Comparison of apoptosis of three MSCs under the stimulation of LPS alone and combination of liraglutide

**Fig. 4:**
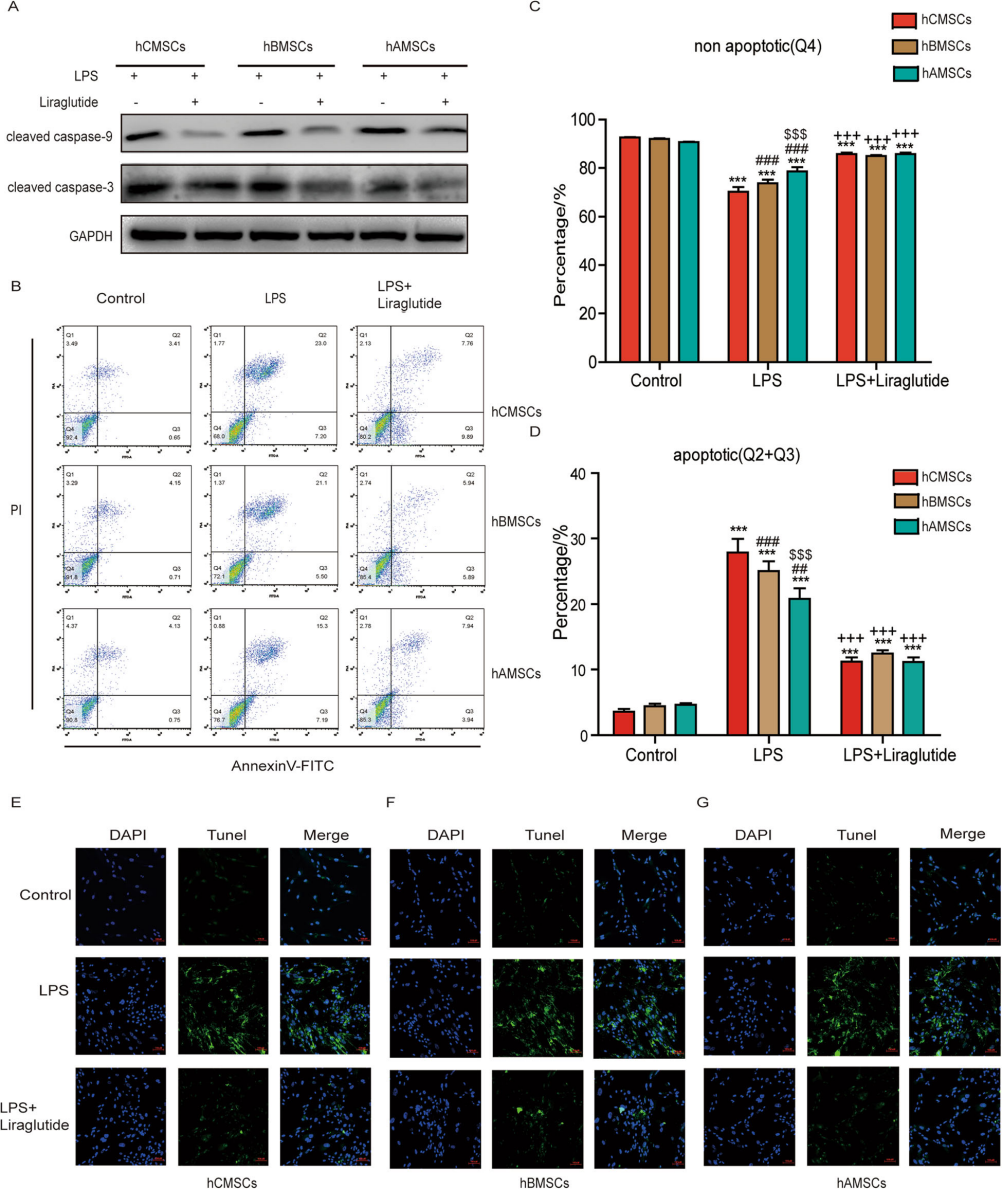
Comparison of the effects of three MSCs on apoptosis under the conditions of LPS alone and combination of liraglutide. **a** Expression of cleaved caspase-9 and cleaved caspase-3 proteins in three MSCs stimulated by 30 μg/mL LPS alone and combination of 10 nM liraglutide. **b** Flow cytometry experiments showed the apoptosis of three MSCs in the control group, LPS group, and LPS + liraglutide group as well as the proportion of no apoptotic cells (**c**) and apoptotic cells (**d**). TUNEL staining images for the apoptosis of hCMSCs (**e**), hBMSCs (**f**), and hAMSCs (**g**) in the control group, LPS group, and LPS + liraglutide group. Blue represented DAPI and green represented protein apoptosis. The representative pictures were showed at × 20 original magnification, scale bars, 100 μm. Error bars represent mean ± SD from three independent experiments. Compared with the control group corresponding to MSCs, ***P < 0.001; compared with the LPS group corresponding to MSCs, ^+++^*P* < 0.001; compared with the corresponding treatment group with hCMSCs, ^##^*P* < 0.01 and ^###^*P* < 0.001; compared with corresponding treatment group with hBMSCs, ^$$$^*P* < 0.001

In order to compare the apoptosis of three MSCs, western blot was used to detect the expression of apoptotic proteins (Fig. 4a). The expression of cleaved caspase-3 and cleaved caspase-9 was increased after LPS stimulation for 72 h, and it reversed by addition of liraglutide. The apoptosis of three MSCs was observed by flow cytometry (Fig. 4b–d). Apoptosis ratio increased significantly after MSCs was cultured in LPS-containing medium for 72 h. hCMSCs were most apoptotic MSCs under the stimulation of LPS. Liraglutide could reduce the percentage of apoptosis. The TUNEL experiment further examined the apoptosis of the three MSCs (Fig. 4e–g). The results were consistent with flow cytometry results. hCMSCs had bright green fluorescence intensity under LPS stimulation.

### Liraglutide inhibits hCMSCs apoptosis through PKA/β-catenin pathway

**Fig. 5:**
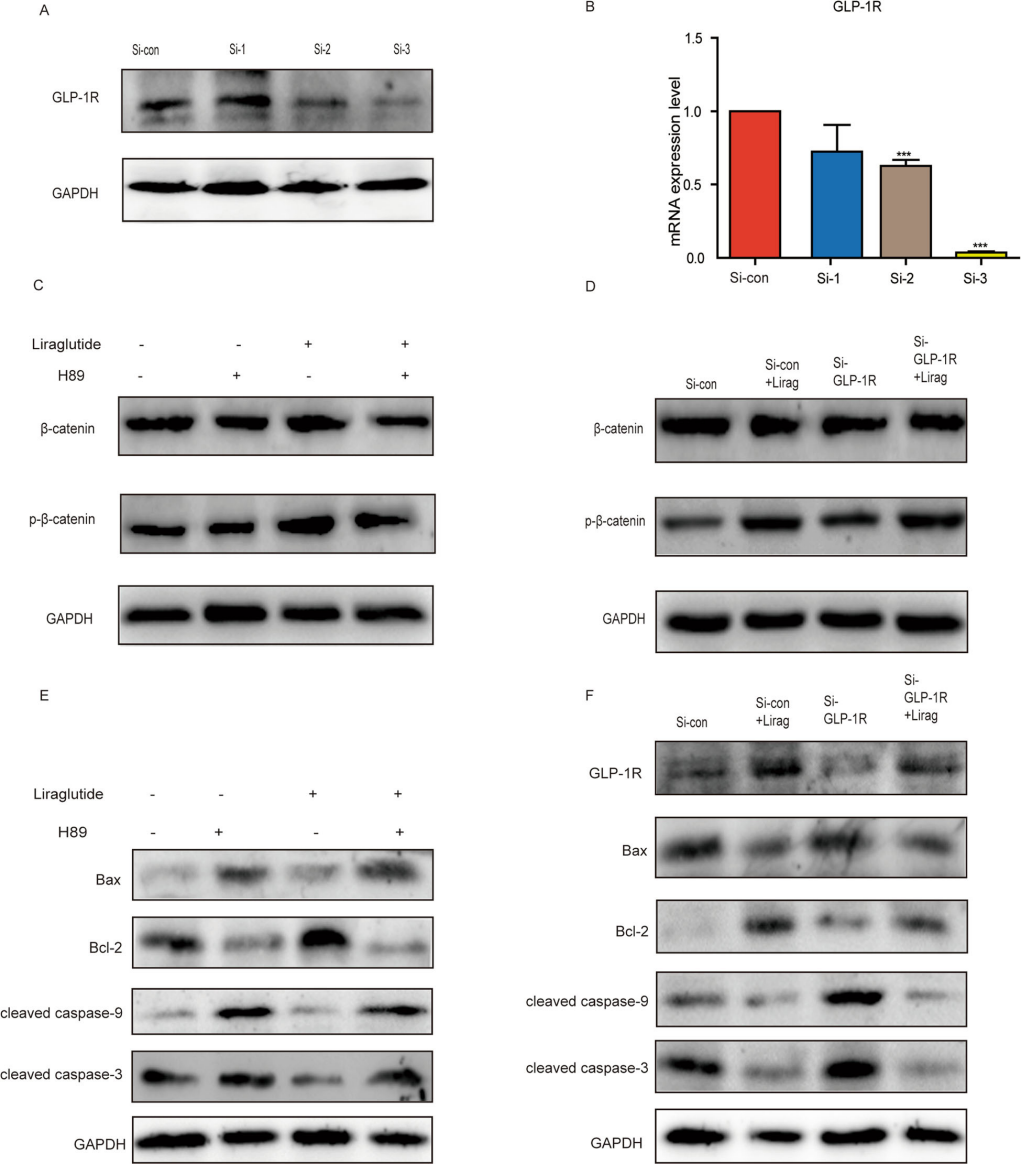
Liraglutide reduces apoptosis of hCMSCs via PKA/β-catenin pathway. **a** Western blot and **b** RT-qPCR verify the knockdown effects of three Si-GLP-1R in hCMSCs. **c**, **d** Western blot was used to detect of β-catenin and p-β-catenin expression under the stimulation of LPS by adding 20 μM H89 or 100 nM Si-GLP-1R and liraglutide. **e** The expression of apoptotic proteins Bax, Bcl-2, cleaved caspase-9, and cleaved caspase-3 was detected by western blot with PKA inhibitor H89 and liraglutide. **f** The expression of GLP-1R and apoptotic proteins Bax, Bcl-2, cleaved caspase-9, and cleaved caspase-3 were detected by western blot with Si-GLP-1R and liraglutide. Error bars represent mean ± SD from three independent experiments. Compared with Si-con group, ***P < 0.001

To certify the mechanism of liraglutide exerting anti-apoptotic function, we selected hCMSCs with obvious apoptosis as our target cells by flow cytometry and TUNEL assay (Fig. 4b, e–g). Since the classical Wnt/β-catenin signaling pathway plays an important role in MSCs, we investigated whether it could participate in the effect of liraglutide on apoptosis of hCMSCs. Liraglutide acted on GLP-1R and we validated the effects of liraglutide by synthesizing exogenous small interfering RNA (SiRNA). Western blot and RT-qPCR were used to analysis of the efficiency of three SiRNA transfections into hCMSCs (Fig. 5a, b), and the Si-3 with the most obvious effect was chosen.

Western blot showed that the expression of phosphorylated β-catenin (p-β-catenin) by adding Si-GLP-1R was attenuated, the expression of Bax, cleaved caspase-9, and cleaved caspase-3 were heightened, and the expression of Bcl-2 was decreased, indicating an increase in apoptosis (Fig. 5d, f).

After the addition of liraglutide, hCMSC apoptosis could be reversed. Simultaneously, we used PKA inhibitor H89 to verify MSC apoptosis. The results showed that H89 inhibited the expression of p-β-catenin and increased the expression of Bax, cleaved caspase-9, and cleaved caspase-3, further reducing the expression of Bcl-2 (Fig. 5c, e). An addition of liraglutide could reduce the apoptosis of hCMSCs.

### The effects of three MSCs alone and combination of liraglutide on acute lung injury in vivo

**Fig. 6:**
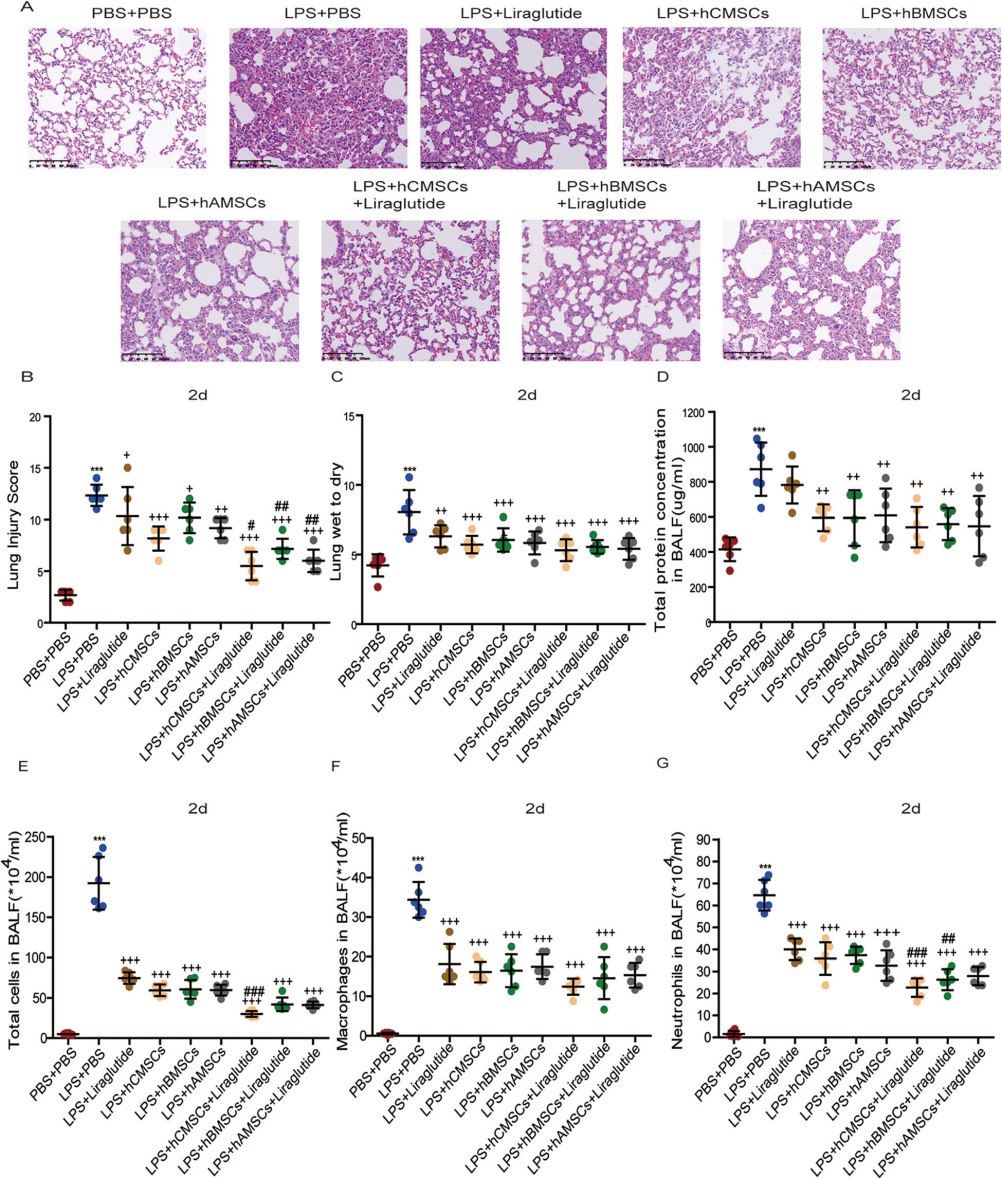
Comparison of the consequence of liraglutide alone, three MSCs alone, and combination of liraglutide in ALI models. **a** The lung tissue sections were observed by H&E staining for histological after 2d of LPS stimulation. The representative sections were showed at × 20 original magnification. **b** Lung injury score, **c** lung wet-to-dry, and **d** total protein concentration in BALF were assessed. **e** Total cells, **f** macrophages, and **g** neutrophils in BALF were assessed by Wright-Giemsa composite dyeing stain counting. Each group contains 6 mice. Error bars represent mean ± SD from three independent experiments. Scale bars, 100 μm. Compared with the PBS + PBS group, ***P < 0.001; compared with the LPS + PBS group, ^+^*P* < 0.05, ^++^*P* < 0.01, and ^+++^*P* < 0.001; compared with the corresponding MSC group, ^#^*P* < 0.05, ^##^*P* < 0.01, and ^###^*P* < 0.001

H&E staining of lung sections was performed for histological evaluation of the ALI models. We observed in 2d model group that in the PBS + PBS group, the pulmonary tissues were arranged neatly and the alveolar cells were uniform in size. Conversely, in the LPS + PBS group, it was evident that the alveolar septum is increased, the alveolar cavities were severely hemorrhagic, and inflammatory cells infiltrated. In the three MSCs alone groups, the degree of lung injury could be alleviated. However, in the liraglutide alone group, the recovery of lung injury was less effective than the MSCs alone. Simultaneously, in the MSCs combined with liraglutide groups, the extent of lung injury was more pronouncedly relieved and the lung injury scores were reduced (Fig. 6a, b). Same trends were also shown in the 7d model group (Additional file 2). And we could conclude that the treatment recovery effect of the 7d model group was preceded than the 2d model group, and hCMSCs combined with liraglutide has a certain advantage in relieving ALI symptoms than the combination liraglutide of hBMSCs or hAMSCs.

Total protein concentration in BALF and lung wet-to-dry ratio are two major indicators for assessing pulmonary edema. In the 2d model group, the total protein concentration and wet-to-dry ratio of the LPS + PBS group were significantly higher than those of the PBS + PBS group. Contrary, the total protein concentration and wet-to-dry ratio were decreased in the three MSCs alone groups and liraglutide alone group. In the three MSCs combined with liraglutide groups, the reduction effect was more obvious. Among them, hCMSCs combined with liraglutide reduced the total protein concentration and wet-to-dry ratio more than hBMSCs and hAMSCs (Fig. 6c, d). The number of total cells, neutrophils, and macrophages in BALF was evidently increased in the LPS + PBS group compared with the PBS + PBS group. Among the three MSCs alone groups and liraglutide alone group, the number of cells lessened. There is significant inflammatory cell reduction in the combination MSCs of liraglutide groups (Fig. 6e–g). The 7d model group also showed the same results (Additional file 3). At the same time, in the 7d model group, the recovery of lung injury was brightly greater than that in the 2d model group. There is significant improvement in acute lung injury symptoms in mice.

### Influence of MSCs alone and combination of liraglutide on the expression of SPC, Ang-1, and KGF in vivo

**Fig. 7:**
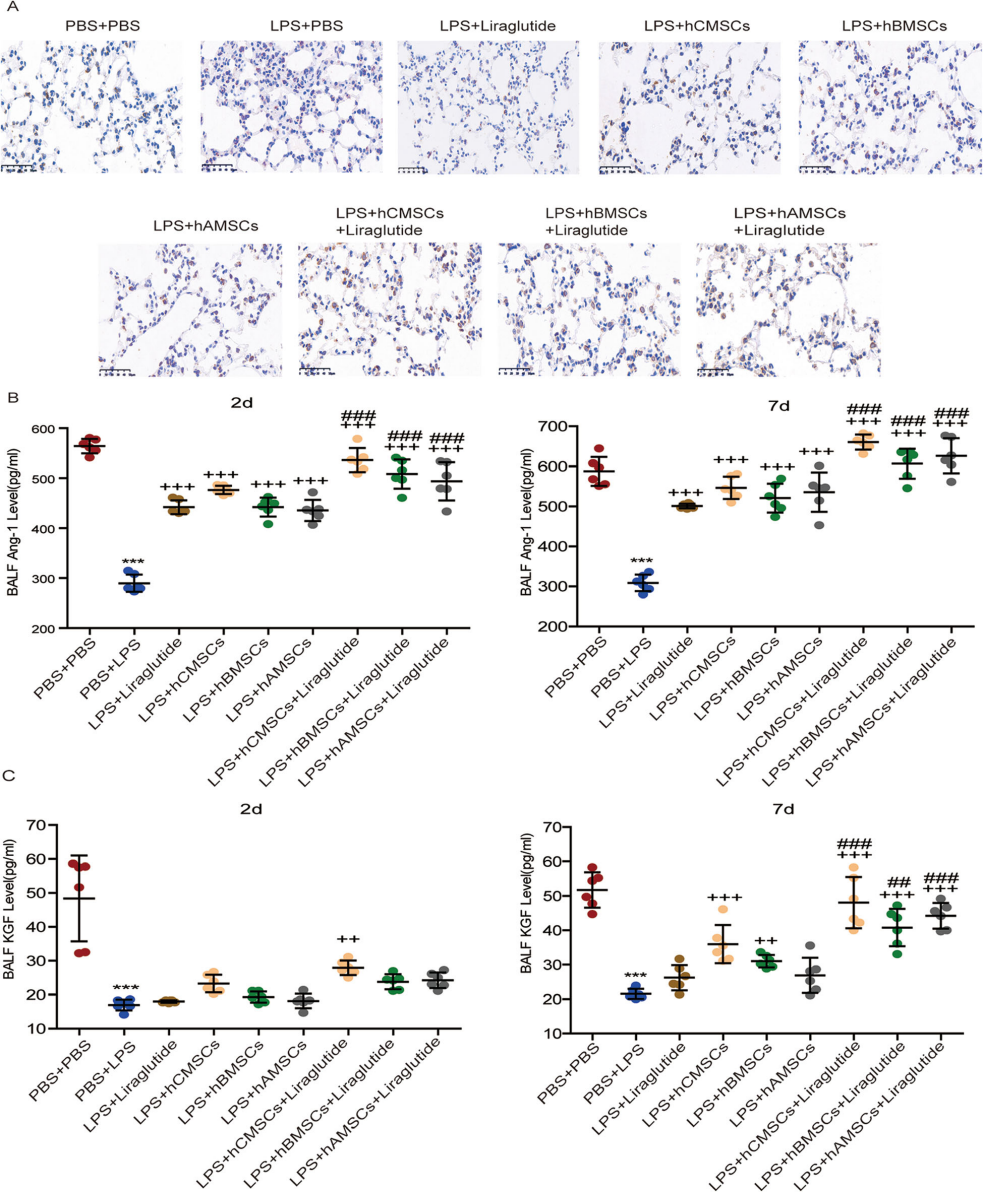
Expression of SPC, Ang-1, and KGF in ALI models. **a** The expression of SPC in lung sections after 7d of LPS stimulation by immunohistochemistry. Brown intracellular precipitation represents SPC protein. The expression of Ang-1 (**b**) and KGF (**c**) in BALF after LPS stimulation for 2d and 7d were detected by ELISA assay. Each group contains 6 mice. Error bars represent mean ± SD from three independent experiments. Scale bars, 50 μm. Compared with the PBS + PBS group, ***P < 0.001; compared with the LPS + PBS group, ^++^*P* < 0.01 and ^+++^*P* < 0.001; compared with the corresponding MSC group, ^##^*P* < 0.01 and ^###^*P* < 0.001

SPC is a protein marker of alveolar type II cells (AEC2s). Immunohistochemistry was used to detect the expression of SPC. In the 7d model group, it was observed that the expression of lung sections is increased in the three MSCs alone groups and liraglutide alone group, and the expression was more notable in three MSCs combined with liraglutide groups (Fig. 7a). In addition, by examining the expression of Nanog protein [35] in the 2d model group, it was found that the expression of Nanog protein increased under the action of liraglutide (Additional file 4). ELISA assay was used to detect the levels of Ang-1 and KGF in BALF. It was found that in the 2d and 7d model groups, the levels of Ang-1 and KGF in the LPS + PBS groups were significantly lower than those in the PBS + PBS groups. Although, the concentration was increased in the three MSCs alone groups and liraglutide alone group, the liraglutide alone group was not as effective as the three MSCs alone groups. The increase was more pronounced in the combination of three MSCs of liraglutide groups; moreover, hCMSCs combined with liraglutide has certain advantages over liraglutide combined with hBMSCs or hAMSCs (Fig. 7b, c).

## Discussion

It was reported in preclinical studies that MSCs from different sources have distinct immunomodulatory and biological functions [13, 14, 16, 18]. hCMSCs belong to the placenta-derived MSCs and are considered to be alternative source of mesenchymal stem cells [36]. Since hCMSCs are readily obtained, they have superior immunomodulatory functions compared with hBMSCs and hAMSCs [37]. Although hBMSCs are widely used adult stem cells, the cell number and multidirectional differentiation potential of these cells were still affected with aging, which limits their availability in the clinical setting [38].. hAMSCs are an emerging candidate for stem cell therapy because of their similar capabilities and differentiation potential to hBMSCs [39]. Some studies have comparatively analyzed the differential properties and biological function of MSCs derived from human chorionic villus, human bone marrow, and human adipose. Although MSCs from different source share similar antigens, their repair potential for ALI is different. Fewer studies have so far investigated their therapeutic effect difference on ALI. In MSC phase I clinical trial, intravenous MSCs was well tolerated in 9 patients with ARDS, and no serious adverse events were reported with MSC administration after 6 months [40]. Subsequently, a double-blind, randomized trial was conducted in a phase II clinical trial, and 60 eligible patients were selected. No side effects associated with MSCs were found in the trial. However, in the experiment, it was found that the survival rate of MSCs is low, only fluctuating between 36 and 85% [41]. Due to the short survival rate of MSCs in the ALI model, we found that combined treatment of MSCs is more evident than MSC alone [42–44].

Previous researches have shown that in addition to being effective in glycemic control, GLP-1 could also perform distinct functions in other tissues [28]. GLP-1 has been shown to exert anti-apoptotic effects in several cells, such as nerve cells [45] and cardiomyocytes [46]. Liraglutide is a long-acting agonist of GLP-1, and it has been reported that liraglutide could inhibit cell apoptosis, including renal tubular epithelial cells [47] and osteoblasts [26]. In our previous research, we also found that MSCs combined with liraglutide can alleviate the inflammation to some extent and reduce the symptoms of ALI [30]. Taking previous studies into account, we explored whether liraglutide could play a role in treatment of ALI by inhibiting MSCs apoptosis.

In this study, we tested the expression of GLP-1R, SPC, KGF, and Ang-1 in hCMSCs, hBMSCs, and hAMSCs under the action of LPS and liraglutide. We found that the expression of Ang-1, SPC, and KGF are enhanced after adding liraglutide. KGF is considered to be an eventful paracrine soluble factor in MSCs and could stimulate the division of ATII in vivo and in vitro [48]. Ang-1 is a key endothelial survival and vascular stabilizing factor. Recent studies have shown that Ang-1 secreted by MSCs could be responsible for inhibiting pulmonary edema and restoring vascular integrity [49]. SPC is a crucial component of pulmonary surfactant and plays an essential role in the regulation of inflammation. Researches have shown that SPC may affect the microenvironment in bronchoalveolar lavage [50, 51].

**Fig. 8:**
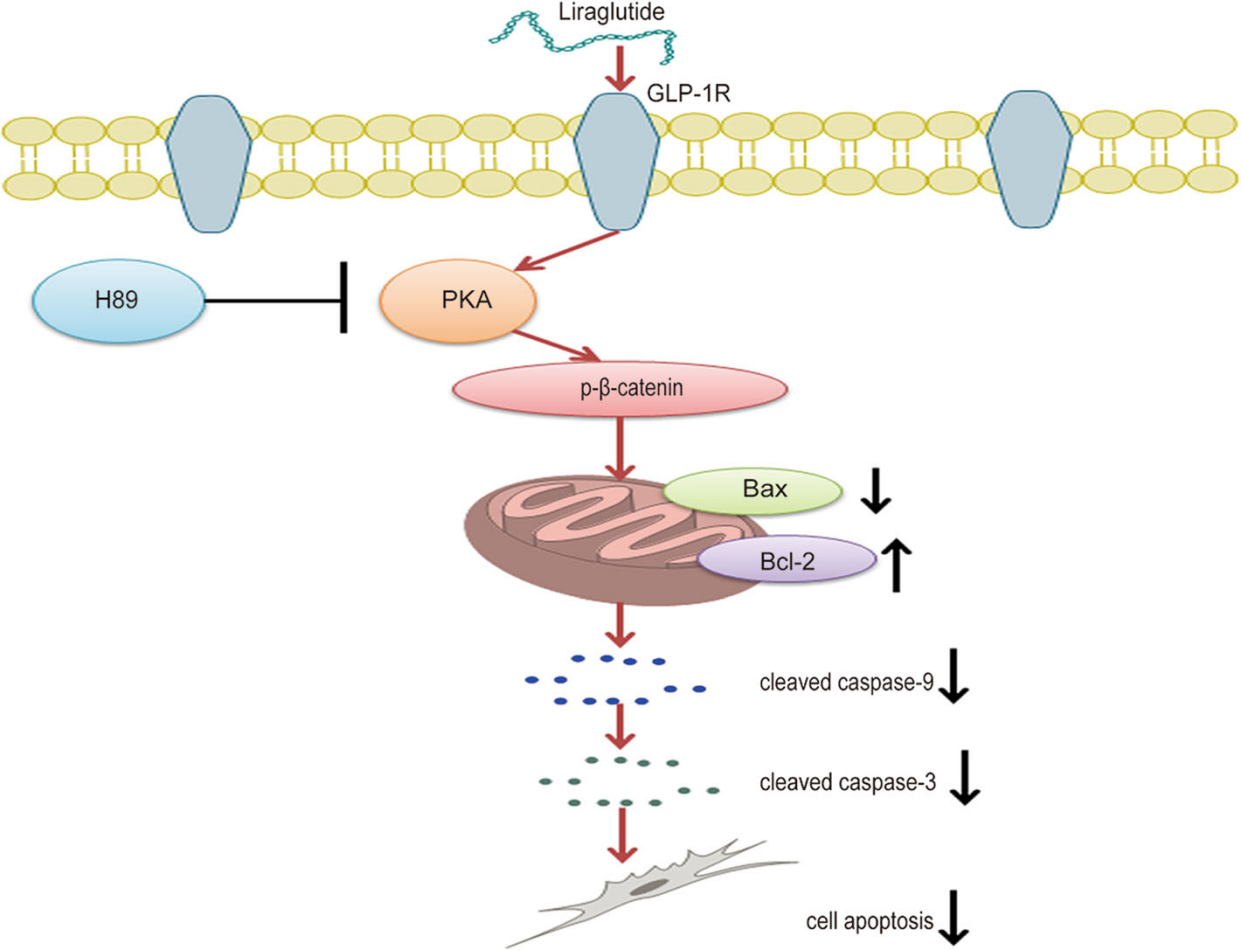
Schematic showing the regulation of apoptosis in MSCs through the PKA/β-catenin pathway. Liraglutide stimulated the expression of PKA by targeting GLP-1R and then motivated the expression of β-catenin, thereby attenuating the expression of Bax, cleaved caspase-9, and cleaved caspase-3 and enhancing the expression of Bcl-2, consequently reducing the apoptosis of MSCs

Hereafter, we detected whether liraglutide could alleviate the symptoms of ALI by reducing MSC apoptosis. Flow cytometry and TUNEL experiments showed that the apoptosis of three MSCs stimulated by LPS is obvious. The addition of liraglutide reversed the apoptosis of MSCs. Then, we verified whether it could inhibit apoptosis by PKA/β-catenin signaling pathway. The expression of apoptotic proteins was examined by the addition of the PKA inhibitor H89 or Si-GLP-1R. Bcl-2 is an anti-apoptotic protein that forms a dimer with proapoptotic protein Bax, which could stimulate the expression of apoptotic protein cleaved caspase-9 and cleaved caspase-3 [52, 53]. H89 and Si-GLP-1R could decrease Bcl-2 expression and increase Bax, cleaved caspase-9, and cleaved caspase-3 expression by inhibiting p-β-catenin expression. Conversely, the addition of liraglutide reversed these results; the expression of Bax, cleaved caspase-9, and cleaved caspase-3 were reduced; and Bcl-2 was increased. The results indicated that liraglutide could reverse LPS-induced apoptosis by restoring the balance of Bcl-2 protein family and caspase protein family (Fig. 8).

In an in vivo experiment with ALI, we compared the liraglutide alone, three MSCs alone, and combination of liraglutide to observe their repair function. In previous studies, MSCs have been shown to repair ALI [13–16, 19], and liraglutide could alleviate the inflammatory environment of ALI [27–29]. In our study, by measuring total protein concentration and cell counts in BALF, wet-to-dry ratio, H&E staining, and lung injury scores, we conclude that liraglutide or MSCs alone and combination of liraglutide could ameliorate the symptoms of ALI. In particular, through the comparison of three MSCs, it was found that hCMSCs combined with liraglutide have a relative advantage in the recovery of symptoms in ALI mice. This trend was also observed in SPC immunohistochemistry experiment as well as the concentration of Ang-1 and KGF. However, the increased expression of SPC in lung tissues may be due to themselves or the secretion of AEC2s stimulated by MSCs. The specific mechanism is unknown, and further investigation is needed.

## Conclusions

Based on our research, we concluded that the effect of MSC combination of liraglutide was prioritized to MSCs or liraglutide alone in the treatment of ALI. Through the PKA/β-catenin signaling pathway, liraglutide could reduce the apoptosis of MSCs. Simultaneously, the degree of lung injury could be ameliorated by increasing the expression of Ang-1, SPC, and KGF, thereby alleviating the symptoms of ALI.

## Supplementary information

**Additional file 1: Figure S1.**The levels of IL-10(A) and TSG-6(B) in culture supernatant. Three kinds of MSCs were stimulated with LPS and Liraglutide for 72 h, then the culture supernatants were collected, and the expression levels of IL-10 and TSG-6 were detected by Elisa assay. Error bars represent mean ± SD from three independent experiments. (Compared with the Control group of corresponding MSCs group, ^***^P<0.001; compared with LPS group of corresponding MSCs group, ^+++^*P*<0.001; compared with LPS+Liraglutide groups of hCMSCs group, ^#^*P*<0.05, ^##^*P*<0.01.).

**Additional file 2.** Comparison of the lung injury therapy of liraglutide alone, three MSCs alone and combination of liraglutide in the 7d ALI models. (A) The lung tissue sections were observed by H&E staining for histological after 7d of LPS stimulation. The representative sections were showed at 20x original magnification. (B) Lung injury score was measured. Scale bars,100 μm. Each group contains 6 mice. Error bars represent mean ± SD from three independent experiments. (Compared with PBS+PBS group, ^***^P<0.001; compared with LPS+PBS group, ^+^*P*<0.05, ^++^*P*<0.01,^+++^*P*<0.001; compared with the corresponding MSCs group, ^#^*P*<0.05, ^##^*P*<0.01).

**Additional file 3.** Comparison of the lung fluid clears and inflammatory cells of liraglutide alone, three MSCs alone and combination of liraglutide in the 7d ALI models. (A) lung wet-to-dry, (B) total protein concentration in BALF were assessed. (C) Total cells, (D) macrophages, (E) neutrophils in BALF were assessed by Wright-Giemsa composite dyeing stain counting. Each group contains 6 mice. Error bars represent mean ± SD from three independent experiments. (Compared with PBS+PBS group, ^***^P<0.001; compared with LPS+PBS group, ^+^*P*<0.05, ^++^*P*<0.01, ^+++^*P*<0.001; compared with the corresponding MSCs group ^#^*P*<0.05, ^###^*P*<0.001).

**Additional file 4.** Immunohistochemistry detected Nanog protein expression in mice lung tissues. The expression of Nanog in lung tissue was detected on the 2d after LPS stimulation. Each group contains 6 mice. Scale bars, 50 μm.

## Data Availability

The data that support the findings of this study are available from the corresponding author upon reasonable request.

## Abbreviations

MSCs: Mesenchymal stem cells
hCMSCs: Human chorionic villus-derived mesenchymal stem cells
hBMSCs: Human bone marrow-derived mesenchymal stem cells
hAMSCs: Human adipose-derived mesenchymal stem cells
ALI: Acute lung injury
ARDS: Acute respiratory distress syndrome
GLP-1: Glucagon-like peptide-1
GLP-1R: GLP-1 receptor
Ang-1: Angiopoietin 1
SPC: SFTPC
BALF: Bronchoalveolar lavage fluid
W/D: Wet-to-dry ratio
SiRNA: Small interfering RNA.
AEC2s: Alveolar type II cells
