# Supplementary figures and images for: Mesenchymal stem cells combined with liraglutide relieve acute lung injury through apoptotic signaling restrained by PKA/β-catenin
Source: Stem Cell Res Ther. 2020 May 19;11:182. doi: 10.1186/s13287-020-01689-5 (PMC7238586; doi:10.1186/s13287-020-01689-5)

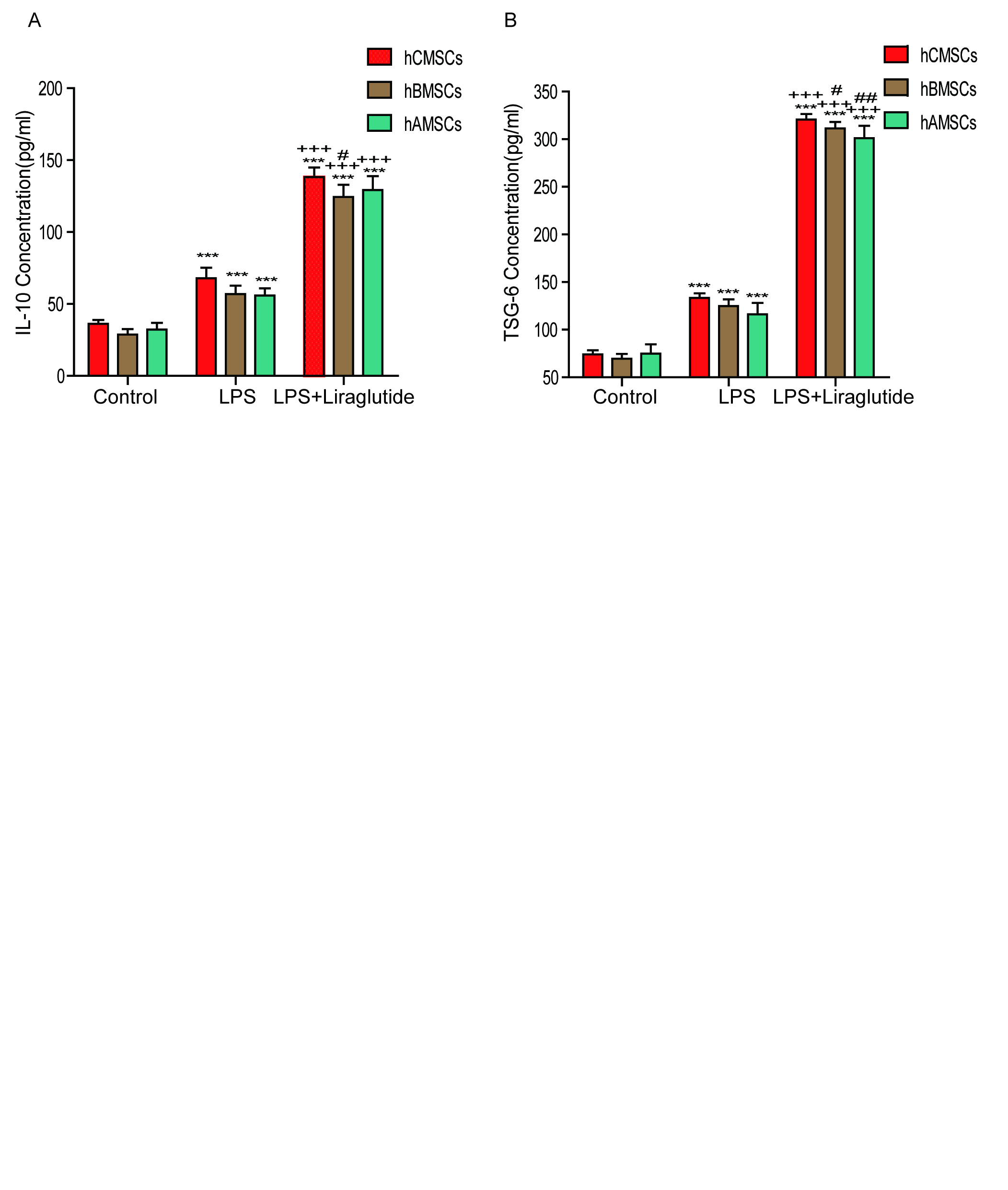

Supplement: Supplementary file 1 — Additional file 1: Figure S1.The levels of IL-10(A) and TSG-6(B) in culture supernatant. Three kinds of MSCs were stimulated with LPS and Liraglutide for 72 h, then the culture supernatants were collected, and the expression levels of IL-10 and TSG-6 were detected by Elisa assay. Error bars represent mean ± SD from three independent experiments. (Compared with the Control group of corresponding MSCs group, ***P<0.001; compared with LPS group of corresponding MSCs group, +++P<0.001; compared with LPS+Liraglutide groups of hCMSCs group, #P<0.05, ##P<0.01.). [file 13287_2020_1689_MOESM1_ESM.tif]

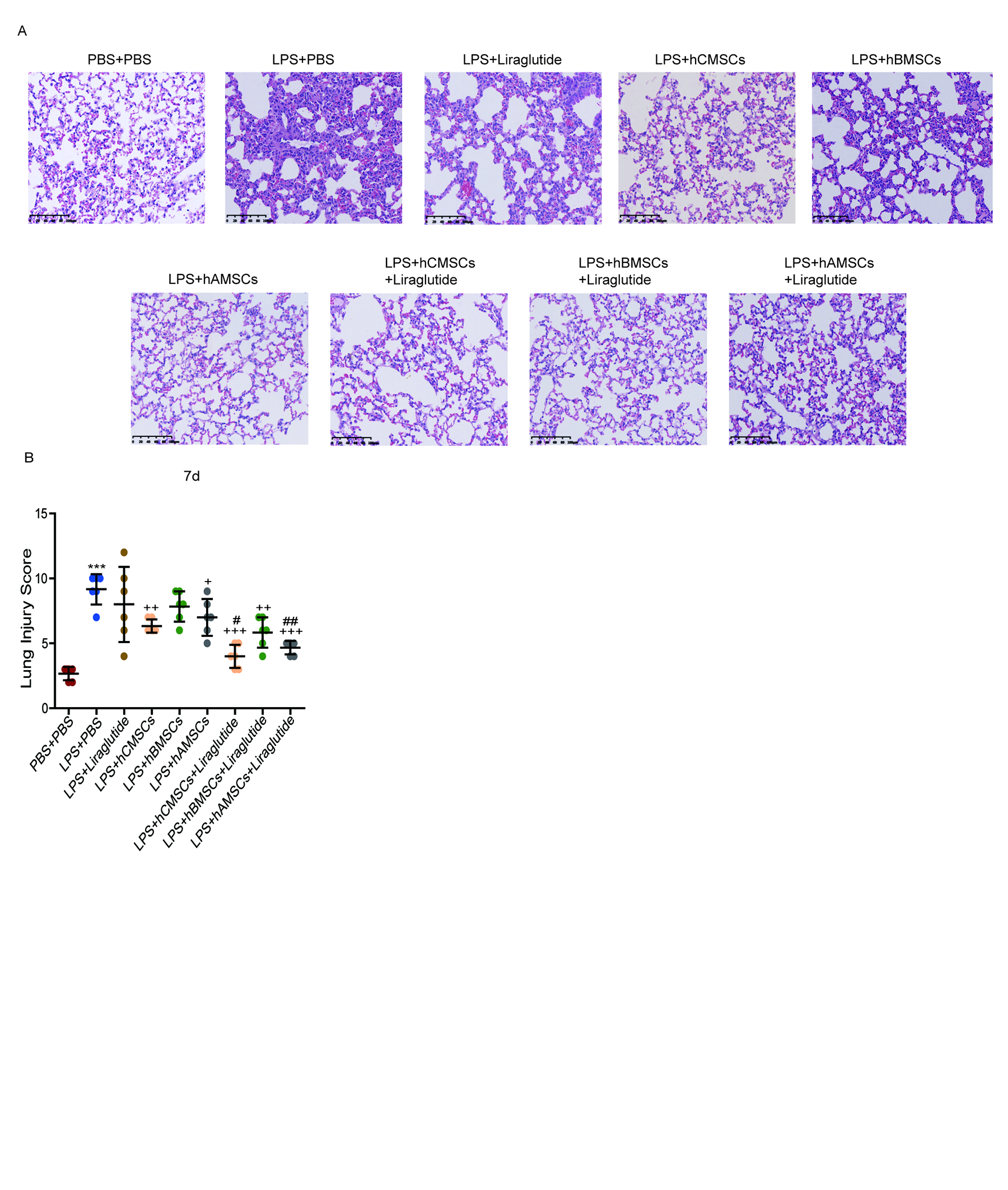

Supplement: Supplementary file 2 — Additional file 2. Comparison of the lung injury therapy of liraglutide alone, three MSCs alone and combination of liraglutide in the 7d ALI models. (A) The lung tissue sections were observed by H&E staining for histological after 7d of LPS stimulation. The representative sections were showed at 20x original magnification. (B) Lung injury score was measured. Scale bars,100 μm. Each group contains 6 mice. Error bars represent mean ± SD from three independent experiments. (Compared with PBS+PBS group, ***P<0.001; compared with LPS+PBS group, +P<0.05, ++P<0.01,+++P<0.001; compared with the corresponding MSCs group, #P<0.05, ##P<0.01). [file 13287_2020_1689_MOESM2_ESM.tif]

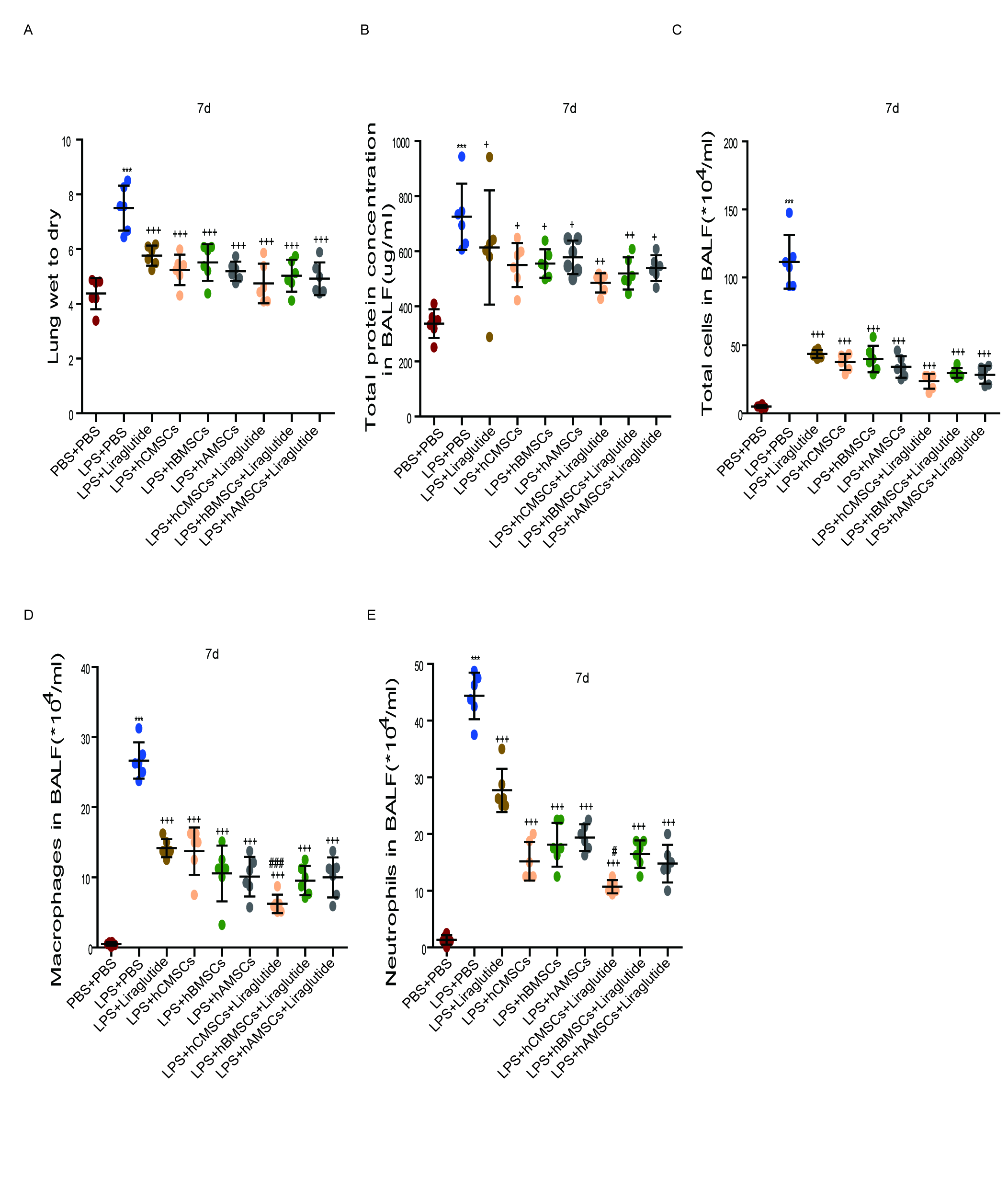

Supplement: Supplementary file 3 — Additional file 3. Comparison of the lung fluid clears and inflammatory cells of liraglutide alone, three MSCs alone and combination of liraglutide in the 7d ALI models. (A) lung wet-to-dry, (B) total protein concentration in BALF were assessed. (C) Total cells, (D) macrophages, (E) neutrophils in BALF were assessed by Wright-Giemsa composite dyeing stain counting. Each group contains 6 mice. Error bars represent mean ± SD from three independent experiments. (Compared with PBS+PBS group, ***P<0.001; compared with LPS+PBS group, +P<0.05, ++P<0.01, +++P<0.001; compared with the corresponding MSCs group #P<0.05, ###P<0.001). [file 13287_2020_1689_MOESM3_ESM.tif]

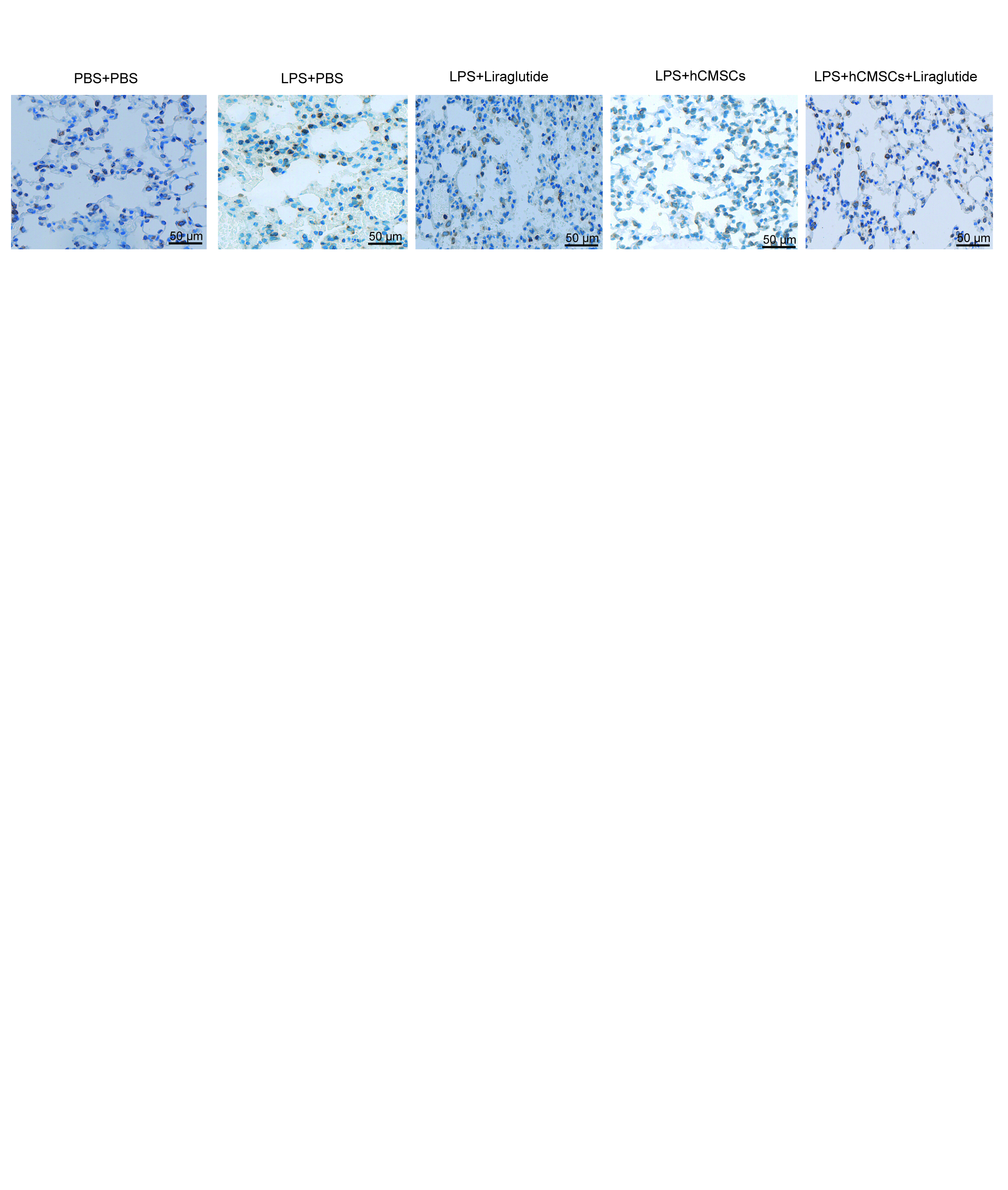

Supplement: Supplementary file 4 — Additional file 4. Immunohistochemistry detected Nanog protein expression in mice lung tissues. The expression of Nanog in lung tissue was detected on the 2d after LPS stimulation. Each group contains 6 mice. Scale bars, 50 μm. [file 13287_2020_1689_MOESM4_ESM.tif]
